# Assessment the properties of various surgical sutures

**DOI:** 10.1038/s41598-025-20311-3

**Published:** 2025-09-29

**Authors:** Doaa H. Elgohary, M. A. Saad, Mona M. Salem, Ehab Haider Sherazy, Tamer F. Khalifa

**Affiliations:** 1 Department of Spinning and Weaving Engineering, Textile Research and Technology Institute, National Research Centre, 33 El Bohouth st. (Former El Tahrir St.), Dokki, P.O.12622, Giza, Egypt; 2 Department of Spinning and Weaving Engineering, Textile Research and Technology Institute, National Research Centre, 33 El Bohouth st., Dokki, P.O.12622, Cairo, Egypt; 3 Department of Spinning and Weaving Engineering, Textile Research Division, National Research Centre, 33 El Bohouth st. (Former El Tahrir St.), Dokki, P.O.12622, Giza, Egypt; 4 Faculty of Applied Arts, Department of Spinning, Weaving and Knitting, Helwan University, P.O.12311, Giza, Egypt; 5 Faculty of Applied Arts, Textile Program, Badr University in Cairo (BUC), P.O.11829, Cairo, Egypt

**Keywords:** Medical, Surgical sutures, Wound healing, Knot tying, Biodegradable yarns, Irritation, Braided sutures, Biotechnology, Engineering, Materials science

## Abstract

The multiple disciplines such as materials science, engineering and biomedicine have facilitated the development of different types of surgical suture materials with multifunctionalities. In this work, thirty-six suture thread material samples were collected from four different companies representing three different materials (most commonly used): silk, VICRYL and polypropylene with three different yarn counts (4, 3.5 And 3 metric). Practical statistical science serves to support the practical analysis of experimental work products and the various relationships between variables to achieve the best sampling performance with the functional purpose generated for it. Analysis of the imported sutures shows that VICRYL sutures had the highest tensile strength, toughness, knot tensile strength and knot toughness, followed by polypropylene and silk. As yarn counts, weight and diameter increase, its tensile strength and toughness increase while its elongation and knot tension decrease. The multifilament yarn construction (silk and VICRYL) scores higher compared to the monofilament construction (polypropylene), resulting in increases in tenacity, toughness, knot tensile strength and knot toughness.

## Introduction

The suture thread was used for wound closure in Egypt as early as 3,500 years BC^1,2^, which considered as an ancient device^3^ and till now suturing is the most common method for promoting tissue healing^1,3^, healing of damaged tissues and organs after surgery^3^.

The choice of suture material and suture method depends on many factors, such as wound configuration and healing time, wound depth, tissue type and the patient’s condition such as age, weight, general health and infection rate^4^., according to Ghosh and Hussey’s report, the ideal suture material should have excellent properties including continuous tensile strength, minimal tissue reactivity, uniform diameter, safe asepsis, easy to absorb tissue exudates, etc^5^. Also it must be easy to handle, form a secure knot, and be biodegradable in a reasonable time when used internally^6,7^. Some surgical sutures that lack biocompatibility tend to cause severe infections or secondary trauma to fragile or soft tissue, so it must be sterile and flexible, exert minimal resistance on the tissue, support abrasion until the growth of new tissue stabilizes the injury site^5,7,8^.

The structure and diameter of the suture material are two parameters associated with adequate tensile resistance. When selecting the size, a balance must be ensured between the size of the suture material and the tissue approximation. This ensures adequate healing^7^. The suture sizes are indicated by a number representing the diameter, ranging in descending order from 10 to 1 And then from 1 − 0 to 12 − 0, where 10 is the largest And 12 − 0 is the smallest diameter than a human hair^9,10^.

Continuing advances in polymer science and technology have given suture materials a variety of options to choose from, expanding the choices from natural extraction to artificial synthesis. Compared to traditional non-degradable suture materials, absorbable materials have some unique properties: they can degrade in vivo without the need for subsequent removal, thereby protecting patients from secondary trauma^3^.

Sutures can be divided into absorbable sutures and non-absorbable sutures. When using non-biodegradable sutures, removal of the sutures is usually necessary. Suture removal is clinically challenging, especially in difficult-to-reach anatomical areas or in pediatric patients. In such cases, the use of biodegradable sutures is recommended^11^.

Absorbable materials break down naturally in the body over time and the byproducts are excreted in the urine. The rate of degradation depends on the material and can take days or even months. Many synthetic suture polymers degrade primarily through hydrolysis of their ester bonds. However, natural polymers such as collagen and silk fibroin are broken down by catalyzed proteolysis^12^, i.e. the breakdown of proteins through the hydrolysis of peptide bonds, which is catalyzed by cellular enzymes called proteases^12,13^. Absorbable suture material easily breaks down into small molecules that can be efficiently excreted from the body due to the presence of water^14,15^, absorbable sutures offer several advantages, including avoiding suture removal, reducing the risk of infection due to the absence of foreign material, and suitability for use in anatomical regions where suture removal is difficult or impossible^16^. Non-absorbable materials are also often used to close skin wounds, although the sutures can be removed after a few weeks^12^.

Silk a natural non-absorbable suture material^17^ that is available in a braided form, as it consists of a cocoon of silkworm larvae^10^. The silk thread consists of two types of proteins fibroin and sericin. Chemically, sericin is a rubbery material used to connect the two triangular filaments of fibroin. Silk threads also contain other natural impurities, namely fat, waxes, inorganic salts and dyes. The composition of proteins and other natural contaminants in Bombyx mori silk^18–20^. Bombyx mori silk has been widely used for making sutures for many centuries. Even the silk protein is a foreign protein to the human body^10^. Silk sutures are coated with different materials such as oil, wax or silicone^10,22^; it has excellent handling characteristics and knot security, as it is mainly used in ophthalmology. The main disadvantages of this suture material are that the coating reduces knot security and causes tissue reaction, infection and capillarity^10^.

Vicryl (polyglactin 910) is an absorbable, synthetic suture that is typically braided. Its perks include increased strength and cosmoses. The disadvantages include sluggish absorption by hydrolysis, increased tissue reactivity, and infection^23^.

Prolene is a non-absorbable synthetic suture with a monofilament structure, the functionalization of their surface to give new properties is of great importance and interest for medicine^23,24^. Its benefits include reduced tissue responsiveness, increased durability, and reduced infection. Polypropylene is immobile and can retain its tensile strength for two years. The tissue reaction is minimal, and the knotting is better than other synthetic sutures^25^. Its disadvantages include fragility, high plasticity, and expensive costs.

## Materials and methods

**Fig. 1:**
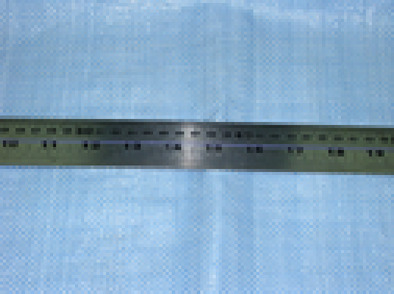
Measuring of Initial Length.

**Fig. 2:**
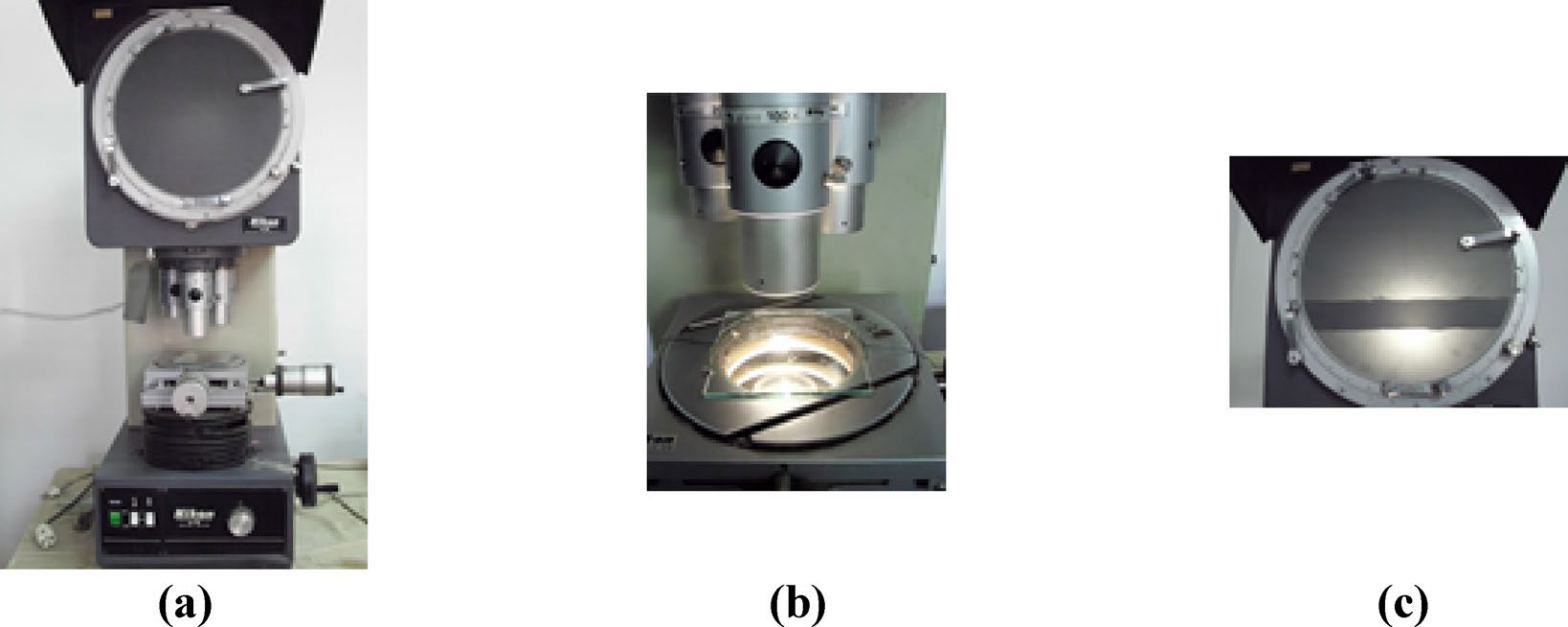
Diameter Measuring Instrument.

**Fig. 3:**
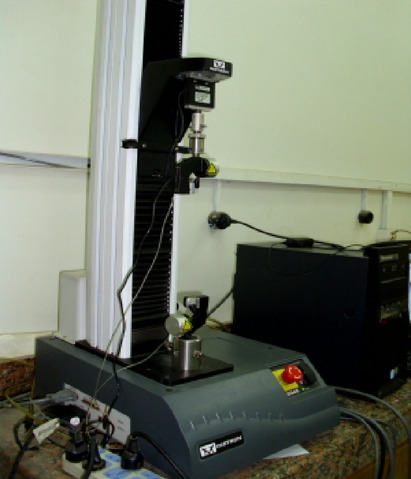
Tensile Strength Instrument.

**Fig. 4:**
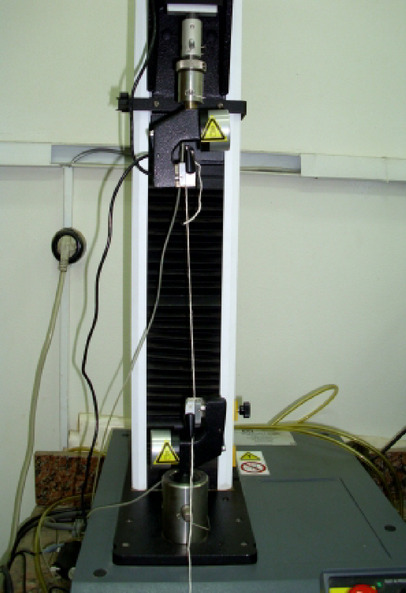
Knot-pull Strength Test.

In this research a comparative study was done between various sutures materials from four different companies. The samples were purchased from four companies (Co.1: Ethicon, Co.2: Tyco, Co.3: Assut Sutures and Co.4:TAISIER.MED). Each company present nine samples, each three samples using three different (silk, VICRYL, polypropylene) material with three different counts (1, 0, 2 − 0) USP, this study depending on the classification of each material according to its length, diameter, weight, yarn count for each yarn Tables 1, 2 and 3. Physical and mechanical properties were done for each sample to investigate its functional efficiency including initial length, Fig. 1, yarn diameter, Fig. 2, yarn tensile strength, Fig. 3, yarn strain, yarn knot-pull strength, Fig. 4, knotting strain and knotting tenacity.

**Table 1:** Samples characterization of silk Sutures.

Company Code	Yarn Count	Yarn Length (cm)	Yarn Weight (g)	Yarn Diameter (mm)
Co.1	1USP (4 Metric)	75	0.18	0.527
	0 USP (3.5 Metric)	75	0.13	0.437
	2/0 USP (3 Metric)	45	0.06	0.389
Co.2	1USP (4 Metric)	180	0.24	0.467
	0 USP (3.5 Metric)	180	0.22	0.393
	2/0 USP (3 Metric)	180	0.17	0.327
Co.3	1USP (4 Metric)	75	0.14	0.486
	0 USP (3.5 Metric)	75	0.11	0.409
	2/0 USP (3 Metric)	75	0.07	0.341
Co.4	1USP (4 Metric)	75	0.13	0.561
	0 USP (3.5 Metric)	75	0.10	0.463
	2/0 USP (3 Metric)	75	0.06	0.391

**Table 2:** Samples characterization of VICRYL Sutures.

Company Code	Yarn Count	Yarn Length (cm)	Yarn Weight (g)	Yarn Diameter (mm)
Co.1	1USP (4 Metric)	75	0.18	0.535
	0 USP (3.5 Metric)	75	0.13	0.43
	2/0 USP (3 Metric)	75	0.09	0.416
Co.2	1USP (4 Metric)	75	0.24	0.527
	0 USP (3.5 Metric)	75	0.15	0.474
	2/0 USP (3 Metric)	75	0.11	0.420
Co.3	1USP (4 Metric)	75	0.16	0.482
	0 USP (3.5 Metric)	75	0.11	0.418
	2/0 USP (3 Metric)	75	0. 09	0.366
Co.4	1USP (4 Metric)	75	0.18	0.512
	0 USP (3.5 Metric)	75	0.13	0.46
	2/0 USP (3 Metric)	75	0.09	0.464

**Table 3:** Samples characterization of polypropylene Sutures.

Company Code	Yarn Count	Yarn Length (cm)	Yarn Weight (g)	Yarn Diameter (mm)
Co.1	1USP (4 Metric)	50	0.08	0.466
	0 USP (3.5 Metric)	100	0.11	0.387
	2/0 USP (3 Metric)	90	0.07	0.322
Co.2	1USP (4 Metric)	75	0.14	0.476
	0 USP (3.5 Metric)	75	0.09	0.387
	2/0 USP (3 Metric)	75	0.06	0.324
Co.3	1USP (4 Metric)	75	0.12	0.475
	0 USP (3.5 Metric)	75	0.07	0.372
	2/0 USP (3 Metric)	75	0.05	0.335
Co.4	1USP (4 Metric)	75	0.11	0.471
	0 USP (3.5 Metric)	75	0.08	0.382
	2/0 USP (3 Metric)	75	0.06	0.326

### Statistical analysis

A Two-way analysis of variance (ANOVA) was used to statistically analysis the effect of yarn materials and yarn count on the mechanical properties, the significant difference was at P-value = 0.05. The difference between means were analyzed using Tukey Honest Significant Difference test at P-value = 0.05. Both tests were analyzed using IBM^®^ SPSS^®^ (SPSS Inc., IBM Corporation, NY, USA) Statistics Version 22 for Windows.

## Results and discussion

Eight properties were measured for thirty-six sutures samples; the data were analyzed according to Two-way Anova statistical analysis and post Tukey test, the results were presented, tabulated and discussed.

### Yarn initial length

**Fig. 5:**
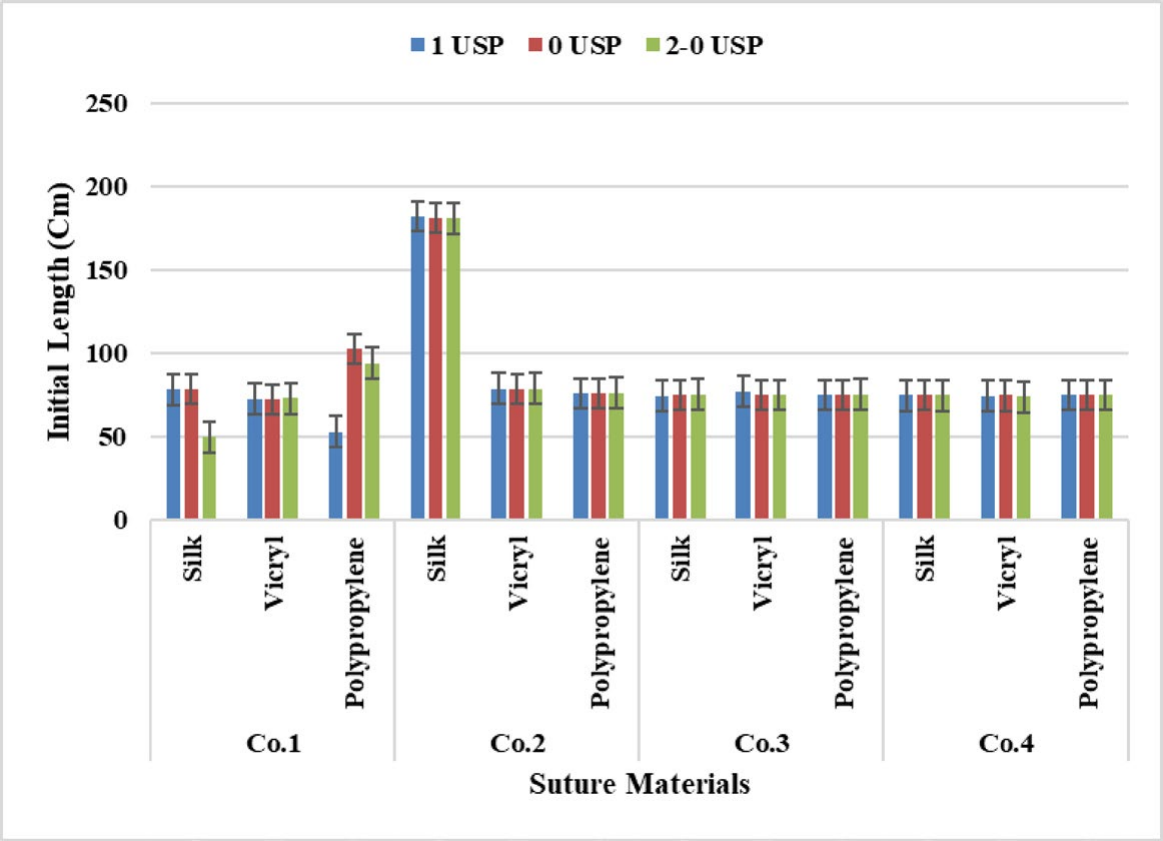
Data Presentation for Initial Length.

Yarn initial length illustrates that by increasing the yarn length the yarn weight increases. This can be interpreted because the increase in length means increasing the fiber length per yarn which leads to increasing the yarn weight, Fig. 5.

From the analysis of two-way Anova Tables 4, 5, 6 and 7, All companies recorded a significant differences for suture material and suture count at P-value (*p* < 0.05), The Tukey Honest Significant Difference test of two-way Anova for all companies presented a significant difference between sutures count and sutures materials at P-value (*p* < 0.05) except Company 3 observed a non-significant value between (Polypropylene – Silk) at P-value (*p* = 0.115) for sutures materials. While for yarn counts a non-significant was recorded between between (3.5–4) at P-value (*p* = 0.521). Furthermore, Company Four for sutures counts a non-significant effect existed between (3–4), (3.5–4) at P-value (*p* = 0.864), (*p* = 0.082) respectively,

### Yarn diameter

**Fig. 6:**
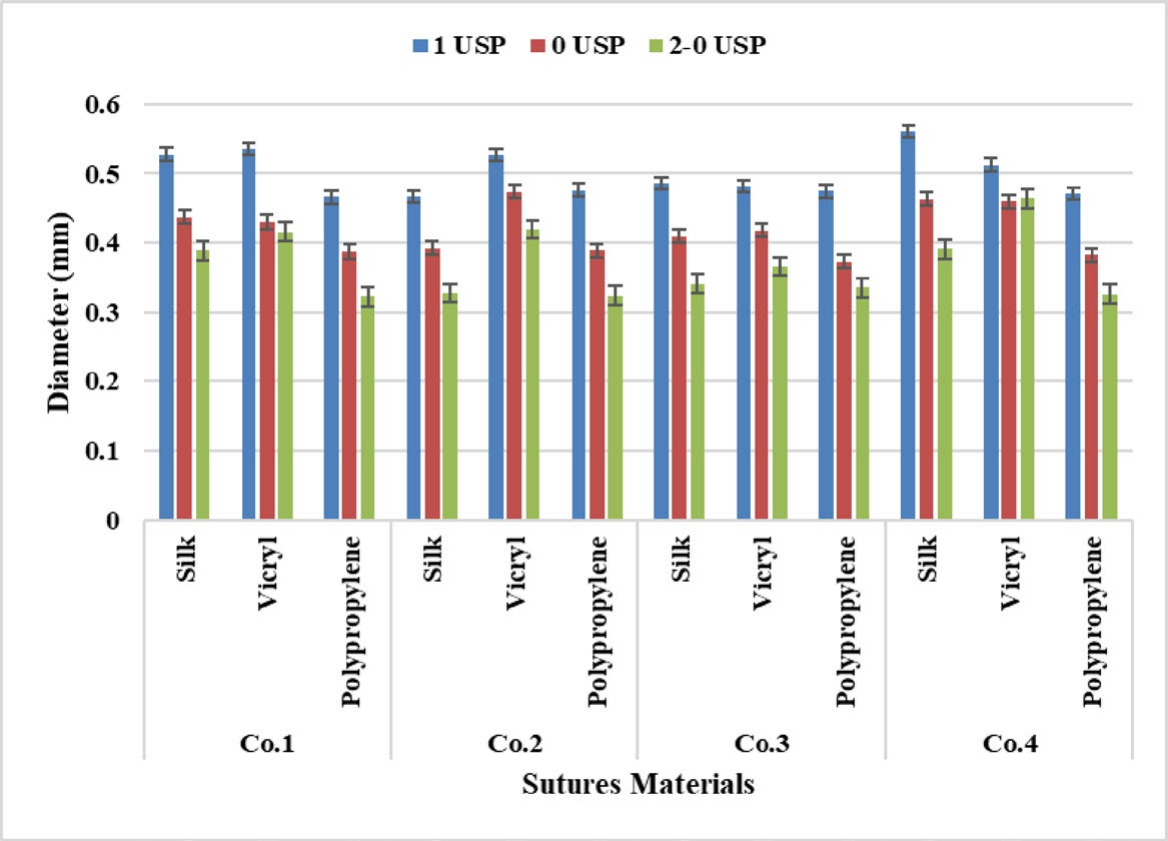
Data Presentation for Diameter.

From the statistical analysis of the results Fig. 6 silk recorded the highest diameter for 4 metric count, while VICRYL recorded the highest score for 3.5 And 3 metric count followed by polypropylene. This can be interpreted due to the thickest yarns have a large number of fibers per unit area for both VICRYL and silk which are multifilament compared with polypropylene which is monofilament.

According to the analysis of Two-way Anova Tables 4, 5, 6 and 7, it was achieved that a significant difference was observed between sutures materials and sutures counts at P-value (*p* < 0.05) for all Companies. Despite for Company four between (Silk – VICRYL) recorded a non-significant effect at P-value (*p* = 0.200). It was shown from the statistical analysis Tukey’s Test for Post-Hoc Analysis of two-way ANOVA that for sutures materials a significant effect occurred for all Companies except between (Silk – VICRYL) recorded a non-significant effect at P-value (*p* = 0.200). Moreover, there is a significant effect presented between all yarn counts at P-value (*p* < 0.05) for all Companies.

### Yarn tensile strength

**Fig. 7:**
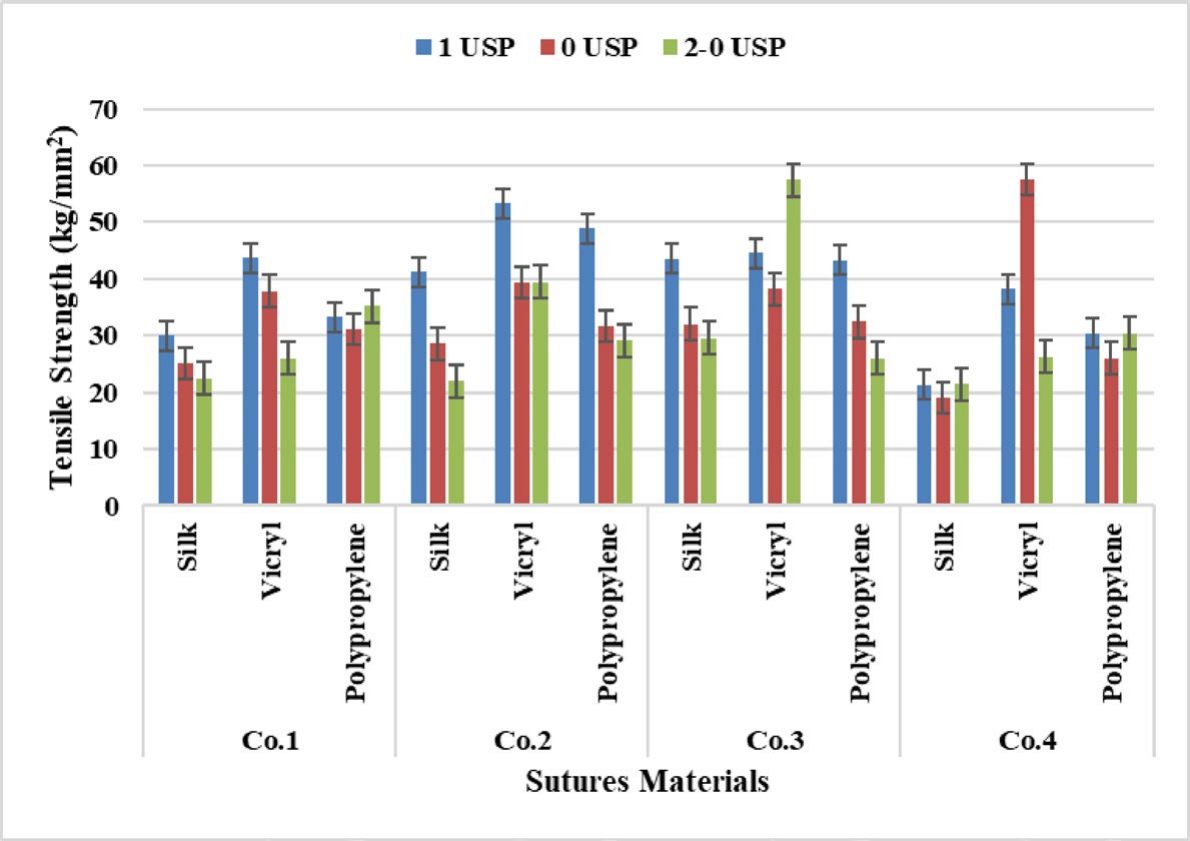
Data Presentation for Yarn Tensile Strength.

It is clear from Fig. 7 that VICRYL has recorded the highest tensile strength followed with polypropylene And silk, according to yarn count, e.g. 4 metric recorded the highest tensile strength compared with 3.5 And 3 metric which recorded the lowest tensile strength gradually. This is due to increasing the number of fibers/yarn which leads to an increase in the tensile strength of the yarn with the largest count.

It was found from the analysis of Anova Tables 4, 5, 6 and 7, that there was a significant effect between samples for sutures materials and sutures count at P-value (*p* < 0.05) for Companies 1, 2, 3, 4. It was demonstrated from Tukey’s Test for sutures materials that a significant effect occurred between all materials for all companies, on the other hand, for sutures count a significant difference presented between all samples at P-value (*p* < 0.05) despite between (3–3.5) and (3.5–4) at P-value (*p* = 0.124), (*p* = 0.054) respectively for Company one, also between (3–3.5) no significant value existed at P-value (*p* = 0.655) for Company two, and between (3.5–4) at P-value (*p* = 0.056) for Company four.

### Yarn strain

**Fig. 8:**
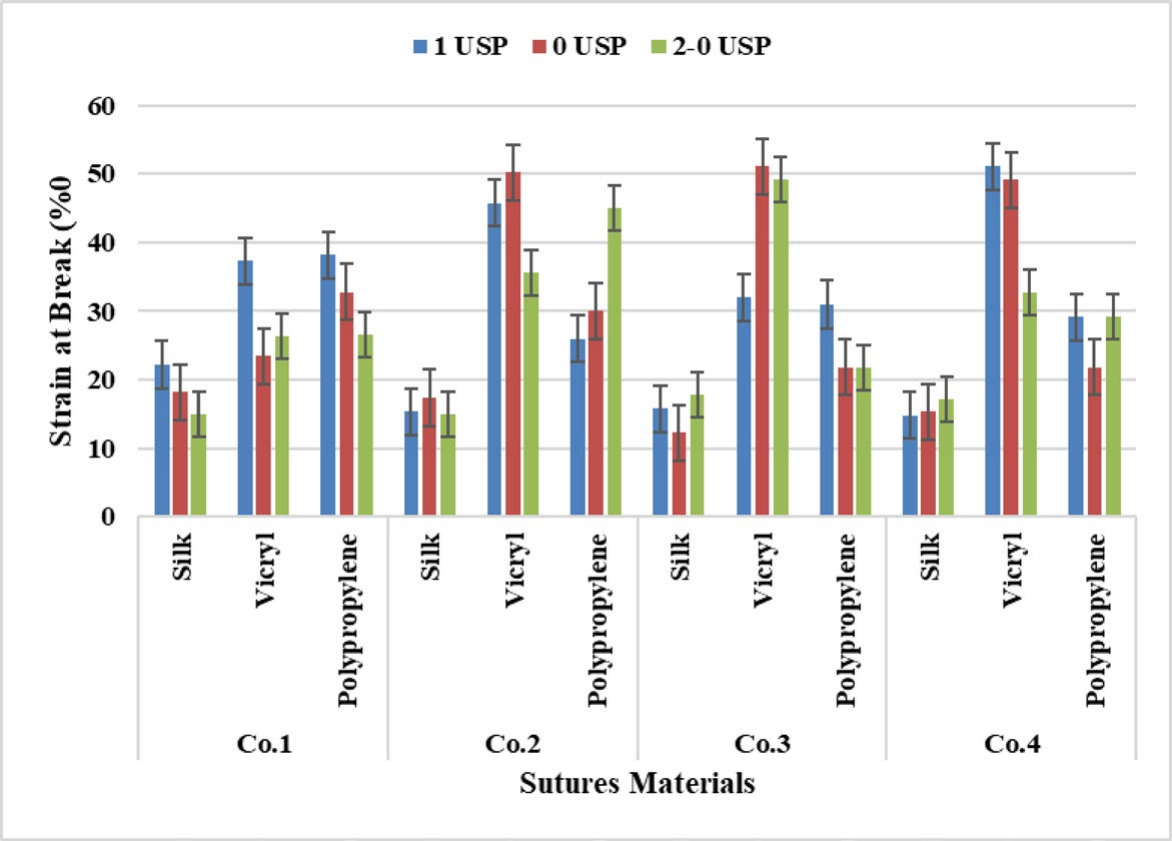
Data Presentation for Yarn Strain at Break.

It was found from the statistical analysis of the results from Fig. 8 that the polypropylene has scored the highest rates of strain followed by VICRYL And silk for the three different counts 4 metric, 3.5 metric And 3 metric gradually. This means that the yarn strain decreases gradually from the large count to less count.

It was demonstrated from the analysis of Two-way Anova Tables 4, 5, 6 and 7 that there was a significant effect between suture counts and sutures materials for all Companies although sutures count for Company four there was no significant difference at P-value (*p* = 0.449). From the analysis of Tukey’s Test for Post-Hoc Analysis of two-way ANOVA, it was revealed that materials recorded a significant effect for all Companies at P-value (*p* < 0.05). Furthermore, a non-significant revealed between (3 −3.5) at P-value (*p* = 0.645) for Company one, between (3.5–4) at P-value (*p* = 0.984) for Company two, between all counts (3–4), (3.5–4) and (3 −3.5) at P-value (*p* = 0.629), (*p* = 0.547) and (*p* = 0.990) respectively for Company three. Moreover, yarn counts recorded no significant effect between (3 −3.5), (3–4) and (3.5–4) at P-value (*p* = 0.581), (*p* = 0.465) and (*p* = 0.979) respectively for Company four.

### Yarn tenacity

**Fig. 9:**
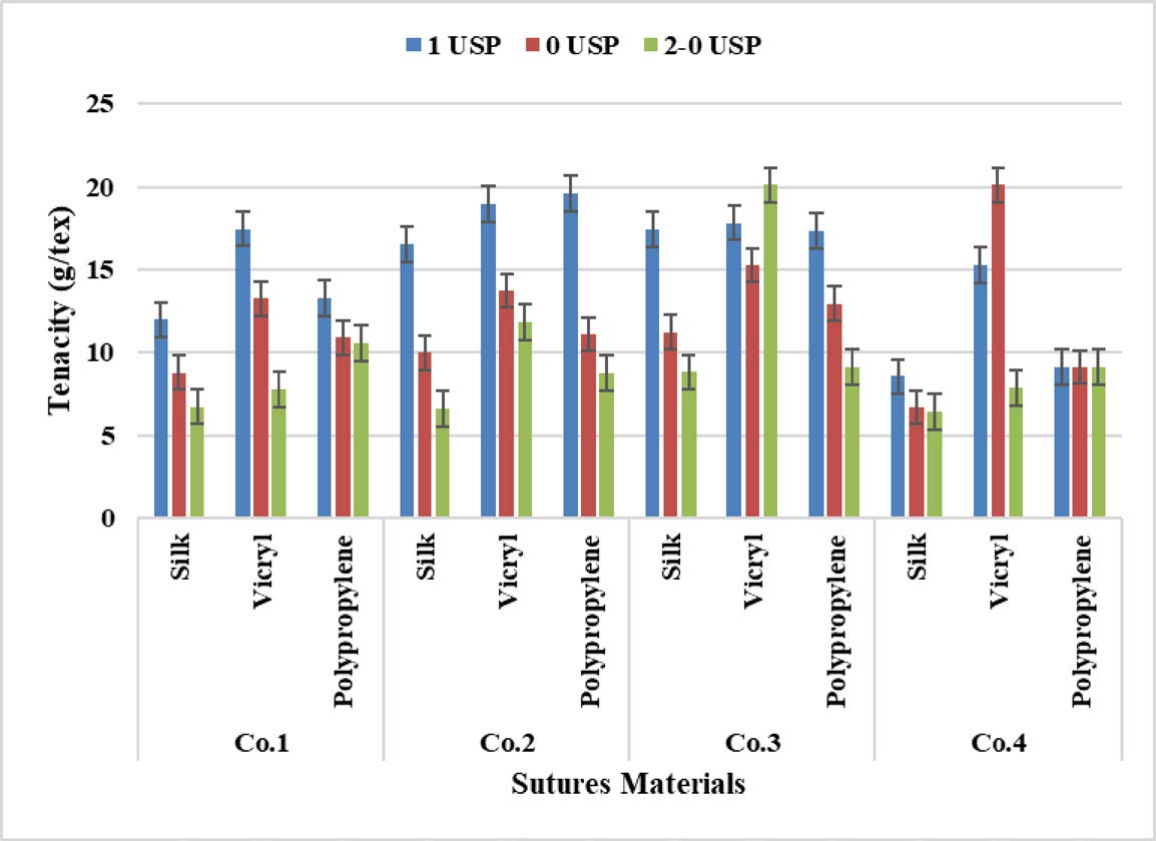
Data Presentation for Yarn Tenacity.

It was found from the statistical analysis of the results from Fig. 9 that VICRYL has recorded the highest score followed by polypropylene and silk, this means that the more yarn weighs the more tenacity, because of increasing the number of fibers per unit area.

It was revealed from the statistical analysis of Two-way ANOVA Tables 4, 5, 6 and 7 there was a significant effect between materials and counts despite materials recorded a non-significant value for Companies three at P-value (*p* = 0.617). From the Tukey Honest Significant Difference test analysis of Two-way Anova there was a significant effect between all materials except between (Polypropylene –VICRYL) at P-value (*p* = 0.106) for Company one, between (Polypropylene – Silk) no significant value occurred at P-value (*p* = 0.559) for Company two, also between (Polypropylene – Silk), (Silk –VICRYL) and Polypropylene –VICRYL) at P-value (*p* = 0.915), (*p* = 0.592) and (*p* = 0.828) respectively for Company three. While for suture counts a significant difference occurred between all counts despite (3 −3.5) counts no significant difference occurred at P-value (*p* = 0.835) for Company four.

### Yarn knot-pull strength

**Fig. 10:**
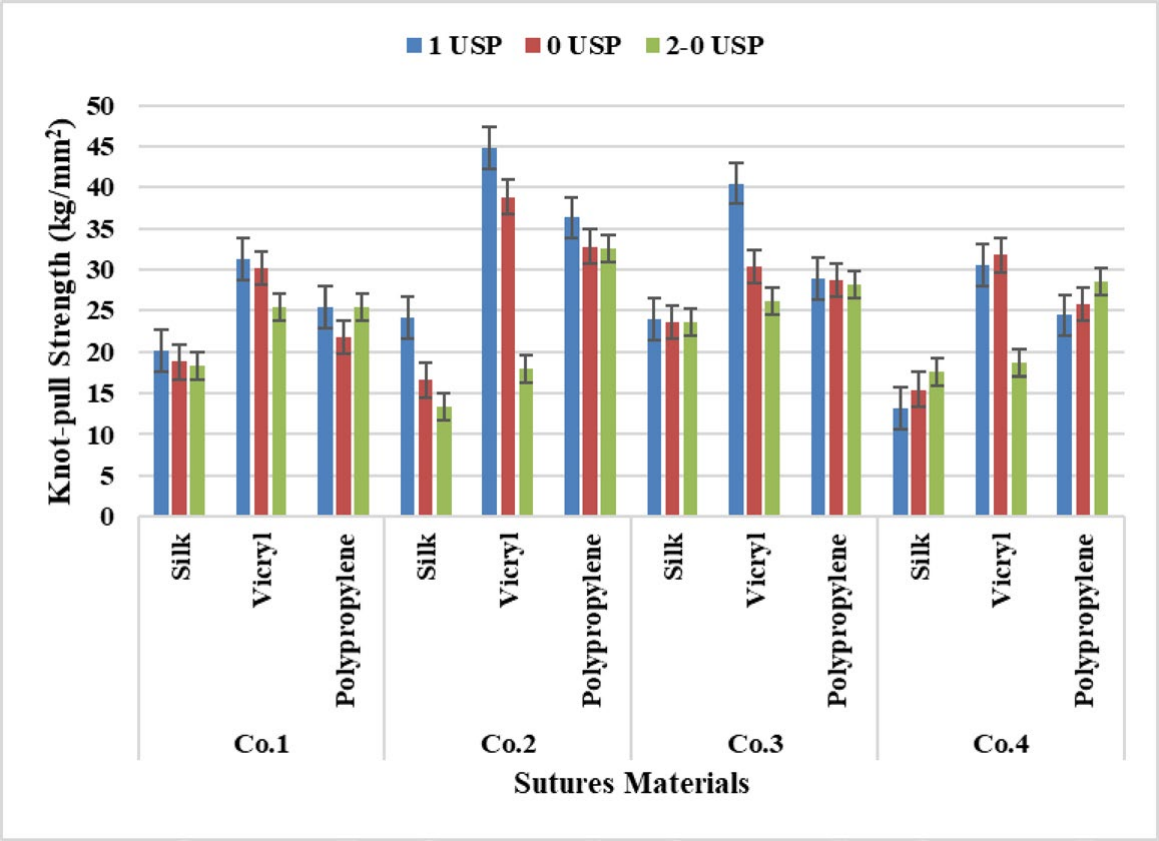
Data Presentation for Yarn Knot-pull Strength.

It is clear from Fig. 10 that VICRYL has recorded the highest tensile strength followed with polypropylene and silk. This is due to increasing the number of fibers/yarn which lead to increase the tensile strength of the yarn also related to the yarn count, so 4 metric recorded the highest knot-pull strength for the three materials followed by the 3.5 And 3 metric counts.

According to analysis of Two-way ANOVA Tables 4, 5, 6 and 7, for suture materials there was a significant difference at P-value (*p* < 0.05), while for sutures count there was no significant effect at P-value (*p* = 0.219), (*p* = 0.131), (*p* = 0.256), for Companies one, three and four. The Tukey test analysis shows a significant difference between all materials although a non-significant occurred between (Polypropylene – Silk), (Polypropylene –VICRYL) recorded non-significant values at P-value (*p* = 0.150), (*p* = 0.303) respectively for Company three, and between (Polypropylene –VICRYL) at P-value (*p* = 0.889) for Company four. On the other hand, for sutures counts all counts recorded a non-significant except Company two recorded a significant value at P-value (*p* = 0.000).

### Yarn knotting strain

**Fig. 11:**
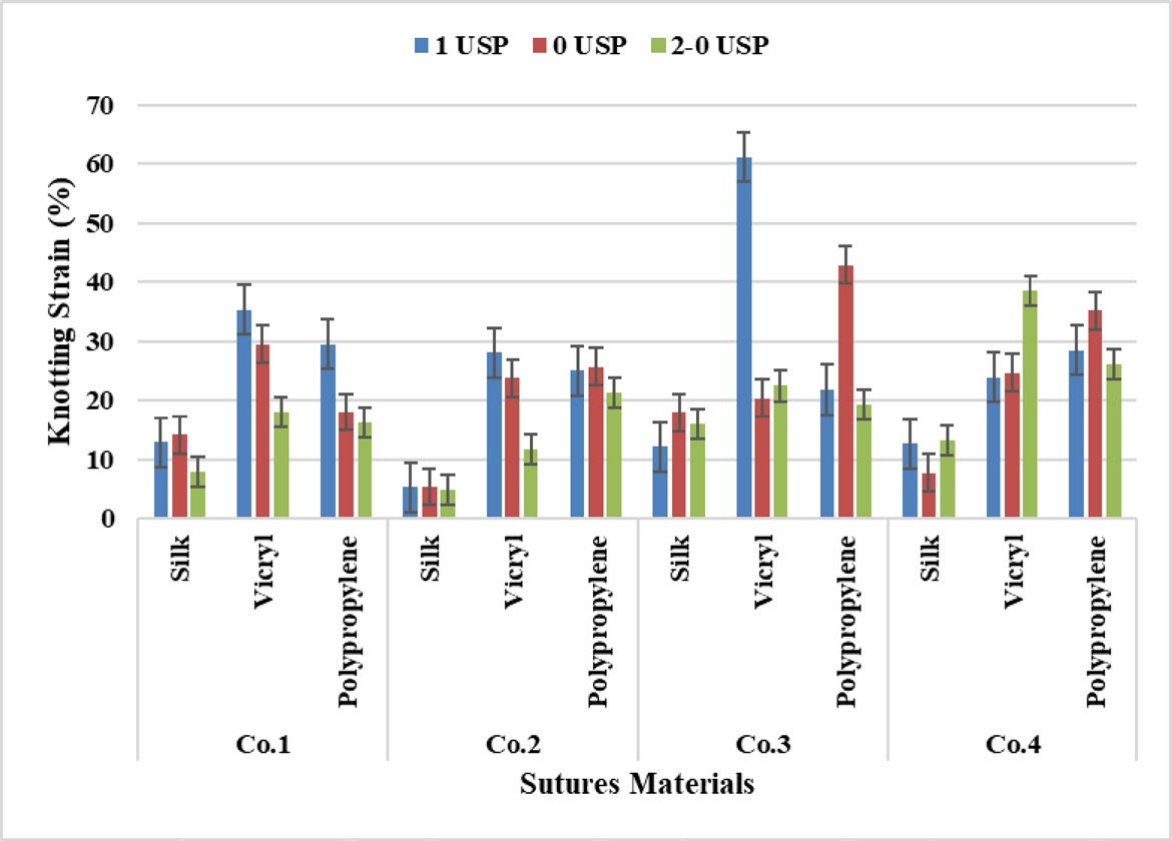
Data Presentation for Yarn Knotting Strain.

It was found from the statistical analysis of the results from Fig. 11 that the VICRYL has scored the highest rates of knotting strain followed by polypropylene And silk for the three different counts 4 metric, 3.5 metric And 3 metric gradually, which means that the yarn strain decreases gradually from the large count to less count.

The analysis of Anova Tables 4, 5, 6 and 7 for yarn materials and yarn counts approved that all materials and counts recorded a significant effect except yarn counts for Company four a non-significant existed at P-value (*p* = 0.128). While Tukey’s Test for Post-Hoc Analysis of two-way ANOVA show a significant difference between all yarn materials and counts despite a non-significant effect occurred for suture count (3.5–4) at P-value (*p* = 0.199) for Company three. Also, no significant difference was exited between (Polypropylene –VICRYL) at P-value (*p* = 0.922), furthermore no significant effect was recorded between (3 −3.5), (3–4), (3.5–4) at P-value (*p* = 0.256), (*p* = 0.135), (*p* = 0.922) respectively for Company four.

### Yarn knotting tenacity

**Fig. 12:**
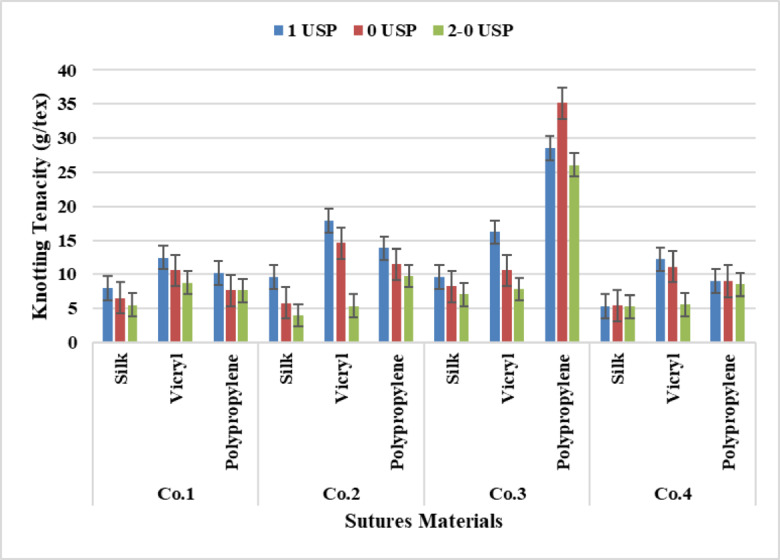
Data Presentation for Yarn Knotting Tenacity.

It is noticed from Fig. 12 that VICRYL has scored the highest rate followed by polypropylene and silk. This is due to the increase of yarn tenacity related to the increase of yarn count which means the increase of fibers per unit area.

The analysis of Two-way Anova Tables 4, 5, 6 and 7 for yarn materials and yarn counts achieved a significant value at P-value (*p* = 0.000) for all Companies. The analysis of the Tukey Honest Significant Difference test for materials observed a significant difference between all suture materials and counts although the non-significant difference between (3–3.5) at P-value (*p* = 0.189) for Company one, between (3–3.5) recorded a non-significant effect at P-value (*p* = 0.114) for Company three. Also, the difference between (3.5–4) showed a non-significant difference at P-value (*p* = 0.559) for Company four. Furthermore, for sutures materials between (Polypropylene –VICRYL) a non-significant value recorded at P-value (*p* = 0.598) for Company four.

**Table 4:** Data analysis for Co.1.

		Sutures Materials				
Variables	Sutures Count	Silk	VICRYL	Polypropylene	Mean	P-value
			Mean ()			
Initial Length (cm)	1USP	78.3(0.408)	72.5(0.147)	53(0.421)	67.933 ^a^	0.000*
	0 USP	78.2 (0.666)	72.1(0.133)	103.1(0.480)	84.467 ^c^	
	2 − 0 USP	49.6 (0.121)	72.8(0.207)	94(0.250)	72.133 ^b^	
Mean		68.7^a^	72.46667 ^b^	83.36667 ^c^		
P-value		0.000*				
Diameter (mm)	1USP	0.527(0.010)	0.535(0.011)	0.466(0.018)	0.509 ^c^	0.000*
	0 USP	0.437 (0.007)	0.43(0.276)	0.387(0.005)	0.418 ^b^	
	2 − 0 USP	0.389(0.016)	0.416(0.013)	0.322(0.009)	0.376 ^a^	
Mean		0.451 ^b^	0.46 ^c^	0.392 ^a^		
P-value		0.000*				
Tensile Strength (kg/mm^2^)	1USP	29.95(1.011)	43.700(2.747)	33.225(1.220)	35.625 ^b^	0.001*
	0 USP	25.18(3.572)	37.850(7.927)	31.13(1.405)	31.38667 ^a, b^	
	2 − 0 USP	22.437(1.286)	25.996(4.658)	35.2(1.976)	27.87767 ^a^	
Mean		25.856 ^a^	35.849 ^b^	33.185 ^b^		
P-value		0.000*				
Strain at Break (%)	1USP	22.20(3.682)	37.25(4.484)	38.15(6.070)	32.533 ^b^	0.001*
	0 USP	18.15(2.656)	23.416(6.88)	32.83(5.937)	24.799 ^a^	
	2 − 0 USP	22.203(5.131)	26.283(5.078)	26.5(3.666)	24.995 ^a^	
Mean		20.851 ^a^	28.983 ^b^	32.493 ^b^		
P-value		0.000*				
Tenacity (g/tex)	1USP)	11.97(0.404)	17.47(1.096)	13.28(0.488)	14.24 ^c^	0.000*
	0 USP	8.80(1.242)	13.24(2.776)	10.89(0.492)	10.977 ^b^	
	2 − 0 USP	6.72(0.382)	7.79(1.402)	10.58(0.593)	8.363 ^a^	
Mean		9.163 ^a^	12.833 ^b^	11.583 ^b^		
P-value		0.000*				
Knot-pull Strength (kg/mm^2^)	1USP	20.074(0.701)	31.21(5.483)	25.407(2.644)	25.564 ^a^	0.219^ns^
	0 USP	18.757(2.868)	30.204(4.027)	21.8(0.586)	23.587 ^a^	
	2 − 0 USP	18.371(2.392)	25.351(3.682)	25.5(1.572)	23.074 ^a^	
Mean		19.067 ^a^	28.921 ^c^	24.236 ^b^		
P-value		0.000*				
Knotting strain (%)	1USP	12.958(3.701)	35.34(2.746)	29.466(4.070)	25.921 ^c^	0.000*
	0 USP	14.133(3.785)	29.496(5.685)	18.1(0.586)	20.576 ^b^	
	2 − 0 USP	7.933(1.103)	18.016(2.332)	16.3(1.193)	14.083 ^a^	
Mean		11.675 ^a^	27.617 ^c^	21.289 ^b^		
P-value		0.000*				
Knotting Tenacity (g/tex)	1USP	8.02(0.280)	12.484(2.193)	10.156(1.057)	10.22 ^b^	0.000*
	0 USP	6.56(1.002)	10.569(1.408)	7.65(0.202)	8.259 ^a^	
	2 − 0 USP	5.51(0.718)	8.77(1.055)	7.65(0.471)	7.31 ^a^	
Mean		6.697 ^a^	10.608 ^c^	8.485 ^b^		
P-value		0.000*				

()-the values in parenthesis indicate the standard deviation.

a-c Means with different superscripts differ(*p* < 0.05), (*) = Significant, (^ns^) = Non-significant.

**Table 5:** Data analysis for Co.2.

		Sutures Materials				
Variables	Sutures Count	Silk	VICRYL	Polypropylene	Mean	P-value
			Mean ()			
Initial Length (cm)	1USP	182.1(0.643)	79(0.163)	75.8(0.345)	112.3 ^a^	0.000*
	0 USP	181.5(0.331)	78.5(0.303)	76(0.261)	112 ^c^	
	2 − 0 USP	181.2(0.187)	79(0.098)	76(0.519)	112.067 ^b^	
Mean		181.6 ^c^	78.833 ^a^	75.933 ^b^		
P-value		0.000*				
Diameter (mm)	1USP	0.467(0.018)	0.527(0.010)	0.476(0.010)	0.49 ^b^	0.000*
	0 USP	0.393(0.014)	0.474(0.008)	0.387(0.012)	0.418 ^a^	
	2 − 0 USP	0.327(0.008)	0.420(0.012)	0.324(0.006)	0.357 ^a^	
Mean		0.396 ^a^	0.474 ^b^	0.396 ^a^		
P-value		0.000*				
Tensile Strength (kg/mm^2^)	1USP	41.3(1.646)	53.256(1.922)	48.93(2.281)	47.829 ^b^	0.000*
	0 USP	28.53(0.473)	39.272(2.877)	31.75(3.419)	33.184 ^a^	
	2 − 0 USP	22(0.608)	39.457(2.320)	29.119(3.624)	30.192 ^a^	
Mean		30.61 ^a^	43.995 ^c^	36.599 ^b^		
P-value		0.000*				
Strain at Break (%)	1USP	15.3(0.289)	45.75(3.688)	26.06(4.015)	29.037 ^b^	0.000*
	0 USP	17.36(0.551)	50.166(4.25)	30.03(5.087)	40.098 ^b^	
	2 − 0 USP	14.93(0.666)	35.583(1.773)	45.066(13.826)	31.859 ^a^	
Mean		15.115 ^a^	43.833 ^c^	33.719 ^b^		
P-value		0.000*				
Tenacity (g/tex)	1USP)	16.52(0.658)	18.97(2.910)	19.57(0.912)	18.353 ^c^	0.000*
	0 USP	9.98(0.163)	13.74(1.006)	11.11(1.201)	11.61 ^b^	
	2 − 0 USP	6.6(0.182)	11.833(0.696)	8.73(1.085)	9.0543 ^a^	
Mean		11.033 ^a^	14.848 ^b^	13.137 ^a^		
P-value		0.000*				
Knot-pull Strength (kg/mm^2^)	1USP	24.1(1.308)	44.770(2.950)	36.302(4.753)	35.057 ^c^	0.000*
	0 USP	16.56(0.666)	38.815(2.218)	32.770(3.580)	29.382 ^b^	
	2 − 0 USP	13.3(1.078)	17.934(4.335)	32.465(6.235)	21.233 ^a^	
Mean		17.987 ^a^	33.839 ^c^	33.845 ^b^		
P-value		0.000*				
Knotting strain (%)	1USP	5.3(0.476)	23.121(5.966)	25.302(2.965)	17.907 ^c^	0.000*
	0 USP	5.34(0.180)	23.779(1.141)	25.749(2.621)	14.559 ^b^	
	2 − 0 USP	4.92(0.715)	11.773(5.393)	21.389(3.457)	12.694 ^a^	
Mean		5.187 ^a^	19.558 ^b^	23.346 ^b^		
P-value		0.000*				
Knotting Tenacity (g/tex)	1USP	9.64(0.523)	17.903(1.180)	13.82(0.776)	13.788 ^c^	0.000*
	0 USP	5.79(0.232)	14.606(2.207)	11.46(1.256)	10.619 ^b^	
	2 − 0 USP	4(0.324)	5.376(1.301)	9.73(1.870)	6.369 ^a^	
Mean		6.476 ^a^	12.628 ^c^	11.67 ^b^		
P-value		0.000*				

()-the values in parenthesis indicate the standard deviation.

a-c Means with different superscripts differ(*p* < 0.05), (*) = Significant, (^ns^) = Non-significant.

**Table 6:** Data analysis for Co.3.

		Sutures Materials				
Variables	Sutures Count	Silk	VICRYL	Polypropylene	Mean	P-value
			Mean ()			
Initial Length (cm)	1USP	74.4(0.957)	77.2(0.366)	75.5(0.075)	75.7 ^b^	0.000*
	0 USP	74.6(1.023)	75(0.737)	75(0.450)	74.867 ^a^	
	2 − 0 USP	75(0.280)	74.9(0.683)	75(0.450)	74.967 ^a^	
Mean		74.667 ^a^	75.7 ^b^	75.167 ^a^		
P-value		0.000*				
Diameter (mm)	1USP	0.486(0.008)	0.482(0.019)	0.475(0.010)	0.481 ^c^	0.000*
	0 USP	0.409(0.012)	0.418(0.013)	0.373(0.008)	0.4 ^b^	
	2 − 0 USP	0.341(0.013)	0.366(0.007)	0.335(0.016)	0.347 ^a^	
Mean		0.412 ^b^	0.422 ^c^	0.394 ^a^		
P-value		0.000*				
Tensile Strength (kg/mm^2^)	1USP	43.511(1.785)	44.589(1.694)	43.286(4.007)	43.795 ^c^	0.000*
	0 USP	32.10(0.777)	42.238(3.066)	37.884(2.017)	37.407 ^b^	
	2 − 0 USP	29.382(3.221)	36.56(1.627)	26.623(2.754)	30.855 ^a^	
Mean		34.998 ^a^	41.129 ^b^	35.931 ^a^		
P-value		0.003*				
Strain at Break (%)	1USP	15.753(1.531)	32(2.66)	30.987(6.722)	26.247 ^a^	0.523^ns^
	0 USP	12.3(0.872)	28.119(8.159)	32.81(1.766)	24.410 ^a^	
	2 − 0 USP	17.766(2.110)	30.16(5.670)	26(4.092)	24.642 ^a^	
Mean		15.273 ^a^	30.093 ^b^	29.932 ^b^		
P-value		0.000				
Tenacity (g/tex)	1USP)	17.40(0.716)	17.83(0.681)	17.31(1.603)	17.51333 ^c^	0.000*
	0 USP	11.23(0.272)	14.78(1.229)	13.28(0.805)	13.097 ^b^	
	2 − 0 USP	8.81(0.858)	10.97(0.569)	7.983(0.962)	8.931 ^a^	
Mean		12.48 ^a^	14.203 ^a^	12.858 ^a^		
P-value		0.617^ns^				
Knot-pull Strength (kg/mm^2^)	1USP	23.938(3.459)	40.484(7.068)	28.836(4.414)	31.086 ^a^	0.131^ns^
	0 USP	23.620(4.611)	30.326(8.216)	28.705(3.451)	27.550 ^a^	
	2 − 0 USP	23.556(5.310)	26.141(4.624)	28.091(3.891)	25.929 ^a^	
Mean		23.705 ^a^	32.317 ^b^	28.544 ^a, b^		
P-value		0.009*				
Knotting strain (%)	1USP	12.101(4.307)	61.04(7.516)	21.835(4.306)	31.659 ^b^	0.000*
	0 USP	17.916(6.481)	20.431(7.419)	42.916(6.002)	27.088 ^b^	
	2 − 0 USP	16.1(0.95)	22.483(4.365)	19.216(3.973)	19.266 ^a^	
Mean		15.372 ^a^	34.651 ^c^	27.989 ^b^		
P-value		0.000*				
Knotting Tenacity (g/tex)	1USP	9.57(1.386)	16.19(2.827)	11.53(3.797)	12.43 ^b^	0.000*
	0 USP	8.263(1.614)	7.84(2.878)	10.043(4.984)	8.715 ^a^	
	2 − 0 USP	7.06(1.591)	7.84(1.385)	8.425(7.203)	7.775 ^a^	
Mean		8.298 ^a^	10.623 ^b^	9.999 ^a, b^		
P-value		0.006*				

()-the values in parenthesis indicate the standard deviation.

a-c Means with different superscripts differ(*p* < 0.05), (*) = Significant, (^ns^) = Non-significant.

**Table 7:** Data analysis for Co.4.

		Sutures Materials				
Variables	Sutures Count	Silk	VICRYL	Polypropylene	Mean	P-value
			Mean ()			
Initial Length (cm)	1USP	75(0.140)	74.2(0.266)	75(0.693)	74.733 ^a, b^	0.022*
	0 USP	75.1(0.13)	74.55(0.055)	75(0.105)	74.883 ^b^	
	2 − 0 USP	75(0.155)	74(0.446)	75(0.141)	74.667 ^a^	
Mean		75.033 ^b^	74.25 ^a^	75 ^b^		
P-value		0.000*				
Diameter (mm)	1USP	0.561(0.023)	0.512(0.020)	0.471(0.013)	0.515 ^c^	0.000*
	0 USP	0.463(0.017)	0.46(0.014)	0.382(0.005)	0.435 ^b^	
	2 − 0 USP	0.391(0.009)	0.464(0.019)	0.326(0.010)	0.394 ^a^	
Mean		0.472 ^b^	0.479 ^b^	0.393 ^a^		
P-value		0.000*				
Tensile Strength (kg/mm^2^)	1USP	21.335(3.828)	38.166(4.597)	32.378(1.765)	30.626 ^b^	0.000*
	0 USP	19.060(3.411)	57.458(1.627)	26.06(2.754)	34.193 ^b^	
	2 − 0 USP	21.404(2.881)	26.068(4.597)	30.430(1.764)	25.967 ^a^	
Mean		20.600 ^a^	40.564 ^c^	29.623 ^b^		
P-value		0.000*				
Strain at Break (%)	1USP	14.483(4.378)	51.076(8.159)	21.847(6.061)	29.135 ^a^	0.449^ns^
	0 USP	15.333(3.482)	49.106(5.670)	21.866(4.092)	28.768 ^a^	
	2 − 0 USP	17.16(6.212)	32.724(2.929)	29.15(6.061)	26.345 ^a^	
Mean		15.659 ^a^	44.302 ^c^	24.288 ^b^		
P-value		0.000*				
Tenacity (g/tex)	1USP)	8.53(1.531)	15.26(1.229)	12.94(0.806)	12.243 ^b^	0.000*
	0 USP	6.66(1.195)	20.10(0.569)	9.116(0.962)	11.959 ^b^	
	2 − 0 USP	6.41(0.862)	7.86(1.377)	9.12(0.528)	7.797 ^a^	
Mean		7.2 ^a^	14.407 ^c^	10.392 ^b^		
P-value		0.000*				
Knot-pull Strength (kg/mm^2^)	1USP	13.166(3.394)	30.521(0.381)	24.430(2.419)	22.706 ^a^	0.256^ns^
	0 USP	15.448(4.208)	31.781(1.622)	25.797(3.496)	24.342 ^a^	
	2 − 0 USP	17.614(3.679)	18.648(3.846)	28.46(5.310)	21.574 ^a^	
Mean		15.409 ^a^	26.983 ^b^	26.229 ^b^		
P-value		0.000*				
Knotting strain (%)	1USP	12.721(3.596)	23.976(0.519)	28.5(3.797)	21.732 ^a^	0.128^ns^
	0 USP	7.768(2.207)	24.909(2.513)	35.15(4.984)	22.609 ^a^	
	2 − 0 USP	13.24(4.071)	38.616(6.754)	26.05(7.203)	25.969 ^a^	
Mean		11.243 ^a^	29.167 ^b^	29.9 ^b^		
P-value		0.000*				
Knotting Tenacity (g/tex)	1USP	5.263(1.359)	12.20(0.155)	9.77(1.228)	9.078 ^b^	0.000*
	0 USP	5.403(1.471)	11.12(0.566)	9.026(1.228)	8.516 ^b^	
	2 − 0 USP	5.28(1.101)	5.586(1.153)	8.53(1.597)	6.465 ^a^	
Mean		5.315 ^a^	9.635 ^b^	9.109 ^b^		
P-value		0.000*				

()-the values in parenthesis indicate the standard deviation.

a-c Means with different superscripts differ(*p* < 0.05), (*) = Significant, (^ns^) = Non-significant.

## Conclusion

Suturing is one of the most common practices in the medical field, as it is a biomaterial device, either natural or synthetic, used to connect blood vessels and connect tissues. The purpose of this study was to provide a scientific study for imported sutures, between different types of sutures from four various companies. Eight properties were measured to determine the efficiency of these threads, from the statistical of sutures it was observed that VICRYL sutures recorded the highest tensile strength, Tenacity, knot-pull strength and Knotting Tenacity followed by polypropylene and silk. With the increase of yarn count, weight, and diameter, its tensile strength and its tenacity increase with the decrease of its strain and knotting strain. Multifilament yarn construction (silk and VICRYL) gives high score compared to monofilament construction (Polypropylene) which lead to increase the tensile strength, Tenacity, Knot-pull strength and Knotting Tenacity. Finally, we can conclude that VICRYL is suitable for internal suturing, where absorbability and tensile strength are important considerations. Instances where polypropylene might still be favored include hernia or vascular repair, which calls for non-absorbable materials. Despite having comparatively lower mechanical metrics, silk is still used in low-tension external closures.

We acknowledge that to improve the findings’ as a clinical relevance, future studies should look at a wider variety of suture types, such as polydioxanone (PDS), chromic catgut, and different types of nylon. To provide a more thorough assessment of suture performance across various clinical scenarios, we advise that these materials be included in future research. Deeper comprehension of the connection between material composition and mechanical and biological behavior.

## Data Availability

All data created, examined or analyzed during this study are included in this published article.
